# Dynamic proline metabolism: importance and regulation in water limited environments

**DOI:** 10.3389/fpls.2015.00484

**Published:** 2015-06-25

**Authors:** Govinal B. Bhaskara, Tsu-Hao Yang, Paul E. Verslues

**Affiliations:** Institute of Plant and Microbial Biology, Academia Sinica, Taipei, Taiwan

**Keywords:** proline, drought, P5CS1, proline dehydrogenase, protein phosphatase 2C, natural variation, post-translational modification, *Arabidopsis thaliana*

## Abstract

Drought-induced proline accumulation observed in many plant species has led to the hypothesis that further increases in proline accumulation would promote drought tolerance. Here we discuss both previous and new data showing that proline metabolism and turnover, rather than just proline accumulation, functions to maintain growth during water limitation. Mutants of Δ*^1^-Pyrroline-5-Carboxylate Synthetase1* (*P5CS1*) and *Proline Dehydrogenase1* (*PDH1*), key enzymes in proline synthesis and catabolism respectively, both have similar reductions in growth during controlled soil drying. Such results are consistent with patterns of natural variation in proline accumulation and with evidence that turnover of proline can act to buffer cellular redox status during drought. Proline synthesis and catabolism are regulated by multiple cellular mechanisms, of which we know only a few. An example of this is immunoblot detection of P5CS1 and PDH1 showing that the Highly ABA-induced (HAI) protein phosphatase 2Cs (PP2Cs) have different effects on P5CS1 and PDH1 protein levels despite having similar increases in proline accumulation. Immunoblot data also indicate that both P5CS1 and PDH1 are subjected to unknown post-translational modifications.

Free proline can accumulate to high levels in drought-stressed plants. For example, tissue proline levels in excess of 100 mM have been reported in the root growth zone of maize seedlings exposed to low water potential (Voetberg and Sharp, 1991; Ober and Sharp, 1994; Verslues and Sharp, 1999). Proline has chemical properties, including high solubility and zwitterionic structure, common to protective compatible solutes (Yancey et al., 1982). Given that proline is likely excluded from the vacuole, even relatively low bulk tissue levels of proline can indicate osmotically significant levels of proline in the cytoplasm and organelles (Bussis and Heineke, 1998). Why proline, rather than other metabolites, accumulates to high levels as well as how proline metabolism may be modified to improve drought tolerance are long standing questions in plant stress biology (Lehmann et al., 2010; Szabados and Savouré, 2010; Verslues and Sharma, 2010; Kavi Kishor and Sreenivasulu, 2014).

Proline is synthesized from glutamate by the action of two enzymes, Δ^1^-pyrroline-5-carboxylate synthetase (P5CS) and Δ^1^-pyrroline-5-carboxylate reductase (P5CR). Conversely, proline catabolism to glutamate occurs via proline dehydrogenase (PDH) and Δ^1^-pyrroline-5-carboxylate dehydrogenase (P5CDH; Szabados and Savouré, 2010; Verslues and Sharma, 2010). Together proline synthesis and catabolism form a cycle the halves of which are separated by compartmentation and, possibly, tissue specific location (Szabados and Savouré, 2010; Verslues and Sharma, 2010). *P5CS1* (*AT2G39800*) and *PDH1* (*AT3G30775*) gene expression patterns suggest that proline synthesis is high and proline catabolism suppressed in photosynthetic tissue during stress while proline catabolism continues at high rate in the root and shoot meristematic regions (Sharma et al., 2011).

Expression of Arabidopsis *P5CS1* is induced by various types of abiotic stress including drought (Savoure et al., 1995; Yoshiba et al., 1995, 1997, 1999; Peng et al., 1996). This, as well as restricted proline accumulation in *p5cs1* mutants, increased proline accumulation of P5CS1 overexpression plants, and study of enzymatic properties of P5CS1 indicated that P5CS1 may be a rate limiting enzyme for proline accumulation (Kavi Kishor et al., 1995; Zhang et al., 1995; Szekely et al., 2008). Reduced expression of Arabidopsis *PDH1* is also thought to be needed for drought-induced proline accumulation (Kiyosue et al., 1996; Yoshiba et al., 1997; Miller et al., 2005; Sharma et al., 2011). *P5CS1* and *PDH1* expression, along with other observations (for example Voetberg and Sharp, 1991; Ober and Sharp, 1994) made it clear that proline metabolism is highly regulated and proline accumulation during drought is not a symptom of stress injury nor a result of passive accumulation caused by growth reduction.

Regulation of proline metabolism under stress has been linked to abscisic acid (Savoure et al., 1997; Strizhov et al., 1997; Abraham et al., 2003); although ABA alone cannot duplicate drought-induced proline accumulation (Sharma and Verslues, 2010). Other data indicate a link of proline metabolism to cellular redox status. Study of P5CR activity found that its regulation by proline and chloride ions differed depending on whether NADH or NADPH was used as the co-factor (Giberti et al., 2014). This observation is consistent with proline metabolism having a special effect on NADP/NADPH ratio (Sharma et al., 2011). Studies of natural variation in proline accumulation also indicate an influence of redox sensitive enzymes including thioredoxins (Verslues et al., 2014) and mitochondrial NAD dehydrogenases (Lovell et al., 2015).

Identification of the key genes in proline metabolism prompted a wave of studies that sought to overexpress *P5CS1* (or its orthologs from other plant species) to increase proline and enhance drought tolerance (for example: Kavi Kishor et al., 1995; Zhu et al., 1998; Sawahel and Hassan, 2002; Su and Wu, 2004; Molinari et al., 2007). Some studies also sought to increase stress tolerance by further suppressing *PDH1* expression (Nanjo et al., 1999; Tateishi et al., 2005). Several studies claimed success in increasing drought tolerance; however, the methods used to evaluate drought tolerance varied greatly and often relied on counting plant survival after rapid severe dehydration rather than on monitoring responses to less severe water limitation which may be more informative (Skirycz et al., 2011; Claeys et al., 2014). Whether or not modification of proline metabolism may be used to engineer drought tolerance, and how such modification should be done, remains uncertain.

The above examples illustrate how experimental design and interpretation have been influenced by the “more is better” view of proline accumulation whereby increasing proline, no matter how it is done, should lead to better drought tolerance. This view is based on the transcriptional up-regulation of *P5CS1* and decreased expression of *PDH1* during drought stress as well as hypotheses that proline turnover under stress is low and that proline accumulation is cell autonomous and isolated from other metabolic pathways (Verslues and Sharma, 2010). It also implies that transcriptional regulation of *P5CS1* and *PDH1* are main determinants of proline accumulation. We propose that these ideas need to be critically examined and present some evidence that support a more dynamic view of proline metabolism during drought and suggest the existence of multiple layers of regulation.

## More is not Always Better: Natural Variation as well as *p5cs1* and *pdh1* Mutants Suggest a More Complex Relationship between Proline Accumulation and Drought Tolerance

Sharma et al. (2011) found that both *p5cs1-4* and *pdh1-2* mutants had similar reductions in growth when transferred from normal media to low water potential PEG-infused agar (–0.7 MPa and –1.2 MPa). Under these conditions, *p5cs1* mutants have reduced proline accumulation while *pdh1* mutants have increased proline, particularly in the root. Exogenous proline could restore growth of *p5cs1* mutants but not *pdh1* mutants, indicating that proline catabolism was required to maintain growth. Furthermore, high *PDH1* expression in meristematic tissue, reduced root tip oxygen consumption in *pdh1-2*, and altered NADP/NADPH all indicated an effect of proline catabolism on redox status and growth (Sharma et al., 2011).

**FIGURE 1:**
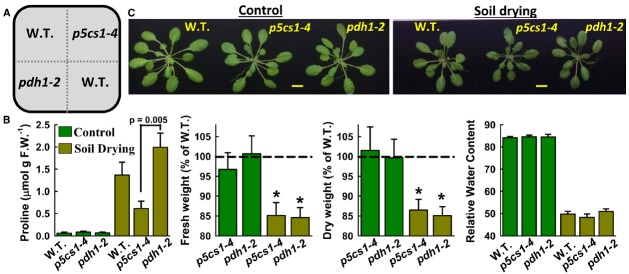
**Mutants of *P5CS1* and *PDH1* have similar growth reductions during soil drying. (A)** Arrangement of genotypes in pots used for soil drying. Two plants were grown in each sector and rosettes harvested at the end of the drying cycle. Twelve to fifteen replicate pots were used for each treatment. **(B)** Proline content, fresh weight, dry weight and relative water content of plants kept under well water conditions or subjected to controlled soil drying. The fresh weight and dry measurements are relative to Columbia wild type, indicated by the dashed line. Asterisks (*) indicate significant differences from wild type by one-sided *t*-test (*p* ≤ 0.05). Error bars indicate standard errors (*n* = 5–9 for proline, *n* = 15–18 for fresh and dry weights and relative water content). Data are combined from two independent experiments. **(C)** Representative rosettes of each genotype from the well watered control and soil drying treatments.

The stress experiments in Sharma et al. (2011) were performed on PEG-infused agar plates. To confirm that these results are applicable to different developmental stages and to drought stress more broadly, we performed controlled soil drying experiments where wild type and mutants were grown together in the same pots to ensure exposure to the same degree of soil drying (Figure 1A). Partial re-watering was performed midway through the drying cycle to equalize water content between the pots and lengthen the exposure to moderate water limitation. Growth data for mutants was normalized to wild type grown in the same pot. Soil water potential was in the range of –0.6 to –0.8 MPa for most of the drying cycle before decreasing to approximately –1.2 MPa by the end of the experiment.

Growth of wild type was reduced approximately 25 percent by the water limitation (data not shown) and proline content increased nearly 20-fold (Figure 1B). Proline accumulation of *p5cs1-4* was less than that of *pdh1-2*; however, both *p5cs1-4* and *pdh1-2* had a similar 15 percent growth reduction in the soil drying treatment (Figures 1B,C). Neither *p5cs1-4* nor *pdh1-2* differed from wild type in the well watered control. There was no difference in relative water content (Figure 1B) indicating that none of the genotypes was more or less dehydrated then the others. The similar growth reduction in *p5cs1-4* and *pdh1-2* despite different levels of proline accumulation argue against the level of proline accumulation itself being the main determinant of drought tolerance. Instead, it may be hypothesized that both *p5cs1-2* and *pdh1-2* have reduced metabolic flux through the cycle of proline synthesis and catabolism and this may be a key factor limiting their growth. We note that proline level in the soil grown plants was less than that of seedlings. This was likely because of the gradual stress imposition and later developmental stage. More mature plants have greater portion of highly vacuolated cells in which proline accumulates in the relatively small volume of the cytoplasm and organelles.

Another set of data relevant to the more-is-better question arises from the 10-fold variation in low water potential-induced proline accumulation among Arabidopsis accessions (Kesari et al., 2012; Verslues et al., 2014). Interestingly, comparing proline accumulation to climate data from accession sites of origin indicated that accessions from generally drier regions had lower proline accumulation (Kesari et al., 2012). Local adaptation is well established in Arabidopsis with accession from dry regions differing in many aspects of their response to water limitation (De Marais et al., 2013; Juenger, 2013). Thus, in the accessions examined so far, adaptation to drier climate seems not to involve increased proline accumulation. This may seem to be at odds with the drought sensitivity of *p5cs1-4* and *pdh1-2*. However, the combined data indicate that while proline accumulation contributes to drought tolerance, accessions that habitually face drought have other metabolic adjustments such that high levels of proline accumulation are not needed. It must also be kept in mind that we do not know if higher or lower proline accumulation correlates with higher or lower flux through proline synthesis and catabolism. As a caveat: the relationship of proline to climate across many accessions is compelling but we recommend due caution in interpretation as the exact microenvironment an accession has adapted to cannot be known and whether some accessions rely on drought escape (such by accelerated flowering at the onset of drought) rather than tolerance of low water potentials is also not clear.

The Shahdara (Sha, also called Shakdara) accession is an interesting example of natural variation in metabolism and drought response. It has been proposed to be a drought tolerant accession (Bouchabke et al., 2008; however, see discussion in Trontin et al., 2011 for questions of Sha’s origin) and is a low proline accumulator mainly because of alternative splicing at the *P5CS1* locus (Kesari et al., 2012). A profile of major metabolites in Sha showed reduced levels of all glutamate family amino acids as well as several major organic acids. In contrast, other amino acids, particularly leucine and isoleucine, had greater drought-induced accumulation in Sha (Sharma et al., 2013). Is this pattern true across a larger number of accessions? Is the lower proline accumulation of Sha indicative of lower (or higher) flux through proline synthesis and catabolism? Do such differences represent a different metabolic strategy of drought tolerance in Sha compared to accessions with higher proline accumulation? Another interesting example is the accession Pt-0 which is essentially a naturally occurring *P5CS1* mutant as it has extreme low levels of P5CS1 transcript and protein and has extreme low level of proline accumulation similar to *p5cs1-4* (Kesari et al., 2012). Is Pt-0 more drought sensitive or does it employ a different metabolic strategy for drought tolerance that makes proline accumulation uneccessary? Answering these questions as well as determining the underlying genetic control of metabolic drought responses is of substantial interest.

## Regulatory Diversity: Protein Phosphatase 2C (PP2C) Mutants Illustrate Multiple Mechanisms Leading to Increased Proline Accumulation

**FIGURE 2:**
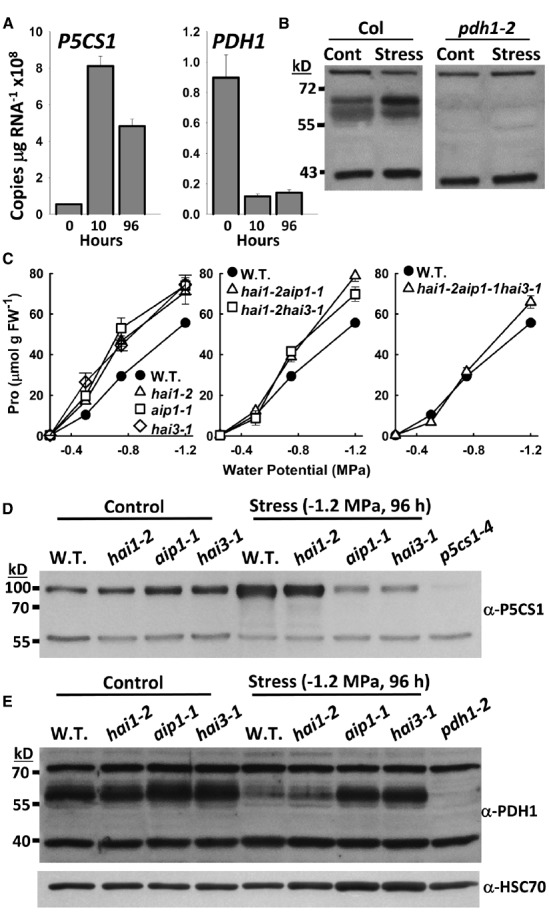
**Immunoblotting reveals differential regulation of P5CS1 and PDH1 protein levels by the Highly ABA Induced (HAI) protein phosphatase 2Cs as well as P5CS1 and PDH1 post-translational modification. (A)** Gene expression of *P5CS1* and *PDH1* at the indicated times after transfer of seedlings from control media (–0.25 MPa) to PEG-infused agar plates (–1.2 MPa). Note that data for seedlings kept at –0.25 MPa was collected but did not show substantial change in expression and has thus been omitted for clarity. Data are re-plotted from Sharma and Verslues (2010). **(B)** Immunoblot detection of PDH1 in seedlings of Columbia wild type or *pdh1-2* under either unstressed conditions (Control) or 24 h after transfer to –1.2 MPa (Stress). All samples were on the same gel and blotted to the same membrane but intervening lanes have been removed for clarity. Non-specific bands at approximately 80 and 40 kD indicate equal loading. **(C)** Proline contents of *hai* mutants at 96 h after transfer to PEG-infused agar plates of a range of low water potential severities. Data are replotted from Bhaskara et al. (2012). **(D)** Immunoblot detection of P5CS1 in Columbia wild type or *hai* mutants under control and stress (–1.2 MPa, 96 h) conditions. An additional lane of *p5cs1-4* (control) was included to verify specificity of the antisera. 50 μg of protein was loaded per lane. The non-specific band at 56 kD indicates equal loading. **(E)** Immunoblot detection of PDH1. The same samples and blotting conditions were used as in D but with *pdh1-2* (grown under control conditions) included to verify antisera specificity. Non-specific bands at 80 and 40 kD indicate equal loading. As an additional check of loading, the blot was stripped and reprobed with anti HSC 70. This blot was also reprobed with anti-P5CS1 which gave the same pattern of P5CS1 protein accumulation as seen in **(D)**.

Transcription of *P5CS1* and *PDH1* is affected oppositely by drought stress in most plant tissues (see example in Figure 2A). While the transcriptional regulation of *P5CS1* and *PDH1* is consistent with accumulation of proline, it is only the first level of regulation. Improvements in proteomics have made it clear that protein levels do not always match transcript levels and the mismatch can be most extreme for transcriptionally down-regulated genes (Vélez-Bermúdez and Schmidt, 2014). This may be the case at least transiently for PDH1 as immunoblots using PDH1 antisera developed in our laboratory show that PDH1 remains high at 24 h after stress treatment (Figure 2B) even though *PDH1* expression was dramatically down regulated by 10 h (Figure 2A). This was consistent with previous observations of PDH1 (Parre et al., 2007). High level of PDH1 present at the same time that proline levels are increasing rapidly have been observed (Kaplan et al., 2007; Schertl et al., 2014) and imply either post-translation regulation of PDH1 activity or sequestering of proline away from PDH1, such as by limited proline transport into the mitochondria. We also previously noted a decrease in *P5CS1* gene expression with no change in P5CS1 protein abundance in *Arabidopsis Histidine Kinase1* (*AHK1*) mutants (Kumar et al., 2013).

Bhaskara et al. (2012), showed that mutants of three Clade A PP2Cs, *Highly ABA-Induced 1* (*HAI1*), *HAI2* (also known as *AKT-Interacting Phosphatase1, AIP1*) and *HAI3*, had increased proline accumulation at low water potential. Curiously, *hai1-2aip1-1* and *hai1-2hai3-1* double mutants had a reduced proline phenotype compared to the single mutants and the high proline phenotype was abolished in a *hai1-2aip1-1hai3-1* triple mutant (Figure 2C; Bhaskara et al., 2012). The reason for this was unclear until we examined P5CS1 and PDH1 protein levels: *aip1-1* and *hai3-1* lack both the low water potential-induced increase in P5CS1 as well as the decrease in PDH1 (Figures 2D,E). Conversely, *hai1-2* has similar P5CS1 and PDH1 protein levels as wild type. Bhaskara et al. (2012) noted that HAI1 had different interaction with the PYL ABA receptors than AIP1 or HAI3, implying a different substrate specificity, and also had substantial effect on gene expression patterns. Thus, we can speculate that HAI1 affects proline accumulation indirectly through changes in regulatory gene expression while AIP1 and HAI2 may affect proline more directly by regulation of P5CS1 and PDH1 expression or protein stability. Possibly, the gene expression changes in *hai1-2* allow wild type levels of proline accumulation even when P5CS1 and PDH1 protein levels are reduced by mutation of *AIP1* or *HAI3*.

The immunoblots indicated that both P5CS1 and PDH1 have unknown post-translational modifications. For P5CS1, its apparent molecular weight of 90–95 kD is heavier than its predicted molecular weight of 77.8 kD (Figure 2D; Kesari et al., 2012). For PDH1, only a small portion of the protein runs at the expected molecular weight of 55 kD while the rest is seen as a broad band or combination of bands from 57 to 65 kD (Figure 2E) similar to previous results (Schertl et al., 2014). The nature of these post-translational modifications is unknown. Redox sensitive modification is one possibility, especially for PDH1 based on its role in electron transport (Servet et al., 2012; Schertl et al., 2014). Other types of modification are possible for P5CS1 where the single band and relatively large shift in molecular weight may be more consistent with sumoylation, glycosylation, or multiple phosphorylation. Whether or not post-translational modification affects P5CS1 or PDH1 activity or localization is of interest for future research.

## Future Perspectives

Several lines of evidence indicate that more proline is not always better for drought tolerance. Rather, the amount of proline that accumulates is dependent on metabolic context and the activity of a number of other metabolic and signaling pathways. How then do we determine the contribution of proline to drought resistance? One point to consider is whether higher or lower proline accumulation is indicative of higher or lower flux through proline synthesis and catabolism and whether the flux and turnover of proline play a key role in drought resistance (see Sharma et al., 2011; Kavi Kishor and Sreenivasulu, 2014 for further discussion). Thus we need to understand the connections of proline metabolism to other metabolic pathways and cellular redox status. Analysis of natural variation through metabolite profiling, and quantitative genetics approaches such as genome wide association analysis and quantitative trait loci mapping can reveal how proline metabolism fits into different drought resistance strategies. The different metabolite profile of Sha discussed above is one example. Also promising are studies of proline metabolism enzymes themselves including localization, which is still unclear for P5CS1, interacting proteins and post-translational modification.

The broad natural variation in proline accumulation indicates that the optimal level of proline accumulation is dependent on species and genotype. This needs to be taken into account in transgenic approaches that seek to modify proline metabolism to improve drought tolerance. Use of stress-inducible promoters may be valuable, but perhaps even more important is to control the tissue specificity of modifications to proline synthesis or catabolism (Sharma et al., 2011). Such experiments should be accompanied by careful analysis of drought physiology, including longer term assays using moderate levels of drought stress where effects of proline metabolism on growth are more relevant to crop productivity and likely to be different than the effects of proline on survival of severe stress.

## Materials and Methods

### Soil Drying Experiments

A standard potting mix was combined with 25% Turface (Turface MVP, Profile Products LLC, USA) to improve porosity and consistency of drying. Seeds of four genotypes were planted in sectors (two plants per sector) of 8 cm × 8 cm × 10 cm (L × W × H) plastic pots (Figure 1A) and grown in a short day chamber (8 h light period, 25 C, light intensity of 100–120 μmol m^–2^ sec^–1^). Hyponex nutrient solution (1 g liter^–1^) was supplied once per week. On 18 day after planting, pots were watered to saturation, allowed to drain and weighed. Water was withheld for 12 days (leading to 50–60 percent reduction in pot weight) and then each pot re-watered to 75 percent of the initial pot weight by injecting water into the middle of the pot with a syringe. The pots were allowed to dry another 8–10 day until pot weight again reached 50–60 percent of the starting weight. Representative rosettes were then photographed and the rest used for measurements of fresh weight, fully hydrated weight and dry weight. Proline was quantified on samples of either whole rosettes (well watered control) or the eighth and ninth leaf (soil drying) using ninhydrin assay (Bates et al., 1973).

## *P5CS1* and *PDH1* Protein Blotting

Seedling growth and stress treatment were performed as previously described (Bhaskara et al., 2012). Protein extraction was carried out as described in Martinez-Garcia et al. (1999) using approximately 100 mg of tissue. Protein contents were measured by BCA assay (Pierce). For each sample, 50 μg of total protein was resolved on 10% SDS PAGE gels and immunoblotting performed serum raised against P5CS1 (Kesari et al., 2012) and PDH1. PDH1 antisera generation and immunoblot procedures were essentially identical to those described in Kesari et al. (2012).

## Author Contributions

PV conceived research and wrote the manuscript with assistance from GB. GB performed all experiments except generation of PDH1 antisera and some immunoblots which were performed by TY. All authors approved the manuscript.

### Conflict of Interest Statement

The authors declare that the research was conducted in the absence of any commercial or financial relationships that could be construed as a potential conflict of interest.

## References

[B1] Abraham E. Rigo G. Szekely G. Nagy R. Koncz C. Szabados L. (2003). Light-dependent induction of proline biosynthesis by abscisic acid and salt stress is inhibited by brassinosteroid in *Arabidopsis*. Plant Mol. Biol. 51, 363–372. 10.1023/A:1022043000516 12602867

[B2] Bates L. S. Waldren R. P. Teare I. D. (1973). Rapid determination of free proline for water stress studies. Plant Soil 39, 205–207. 10.1007/BF00018060

[B3] Bhaskara G. B. Nguyen T. T. Verslues P. E. (2012). Unique drought resistance functions of the Highly ABA-Induced clade A protein phosphatase 2Cs. Plant Physiol. 160, 379–395. 10.1104/pp.112.202408 22829320 PMC3440212

[B4] Bouchabke O. Chang F. Q. Simon M. Voisin R. Pelletier G. Durand-Tardif M. (2008). Natural variation in *Arabidopsis thaliana* as a tool for highlighting differential drought responses. PLoS ONE 3:e1705. 10.1371/journal.pone.0001705 18301780 PMC2246160

[B5] Bussis D. Heineke D. (1998). Acclimation of potato plants to polyethylene glycol-induced water deficit. II. Contents and subcellular distribution of organic solutes. J. Exp. Bot. 49, 1361–1370. 10.1093/jxb/49.325.1361

[B6] Claeys H. Van Landeghem S. Dubois M. Maleux K. Inze D. (2014). What is stress? Dose-response effects in commonly used *in vitro* stress assays. Plant Physiol. 165, 519–527. 10.1104/pp.113.234641 24710067 PMC4044843

[B7] De Marais D. L. Hernandez K. M. Juenger T. E. (2013). Genotype-by-environment interaction and plasticity: exploring genomic responses of plants to the abiotic environment. Annu. Rev. Ecol. Evol. Syst. 44, 5–29. 10.1146/annurev-ecolsys-110512-135806

[B8] Giberti S. Funck D. Forlani G. (2014). *Δ*^1^-pyrroline-5-carboxylate reductase from *Arabidopsis thaliana*: stimulation or inhibition by chloride ions and feedback regulation by proline depend on whether NADPH or NADH acts as cosubstrate. New Phytol. 202, 911–919. 10.1111/nph.12701 24467670

[B9] Juenger T. E. (2013). Natural variation and genetic constraints on drought tolerance. Curr. Opin. Plant Biol. 16, 274–281. 10.1016/j.pbi.2013.02.001 23462639

[B10] Kaplan F. Kopka J. Sung D. Y. Zhao W. Popp M. Porat R. (2007). Transcript and metabolite profiling during cold acclimation of *Arabidopsis* reveals an intricate relationship of cold-regulated gene expression with modifications in metabolite content. Plant J. 50, 967–981. 10.1111/j.1365-313X.2007.03100.x 17461790

[B11] Kavi Kishor P. B. Hong Z. Miao G. H. Hu C. A. A. Verma D. P. S. (1995). Overexpression of *Δ*^1^-pyrroline-5-carboxylate synthase increases proline production and confers osmotolerance in transgenic plants. Plant Physiol. 108, 1387–1394. 12228549 10.1104/pp.108.4.1387 PMC157516

[B12] Kavi Kishor P. B. Sreenivasulu N. (2014). Is proline accumulation per se correlated with stress tolerance or is proline homeostasis a more critical issue? Plant Cell Environ. 37, 300–311. 10.1111/pce.12157 23790054

[B13] Kesari R. Lasky J. R. Villamor J. G. Marais D. L. D. Chen Y. J. C. Liu T. W. (2012). Intron-mediated alternative splicing of *Arabidopsis* P5CS1 and its association with natural variation in proline and climate adaptation. Proc. Natl. Acad. Sci. U.S.A. 109, 9197–9202. 10.1073/pnas.1203433109 22615385 PMC3384178

[B14] Kiyosue T. Yoshiba Y. Yamaguchi-Shinozaki K. Shinozaki K. (1996). A nuclear gene encoding mitochondrial proline dehydrogenase, an enzyme involved in proline metabolism, is upregulated by proline but downregulated by dehydration in *Arabidopsis*. Plant Cell 8, 1323–1335. 10.1105/tpc.8.8.1323 8776899 PMC161248

[B15] Kumar M. N. Jane W. N. Verslues P. E. (2013). Role of the putative osmosensor *Arabidopsis* histidine kinase1 in dehydration avoidance and low water potential response. Plant Physiol. 161, 942–953. 10.1104/pp.112.209791 23184230 PMC3561031

[B16] Lehmann S. Funck D. Szabados L. Rentsch D. (2010). Proline metabolism and transport in plant development. Amino Acids 39, 949–962. 10.1007/s00726-010-0525-3 20204435

[B17] Lovell J. T. Mullen J. L. Lowry D. B. Awole K. Richards J. H. Sen S. (2015). Exploiting differential gene expression and epistasis to discover candidate genes for drought-associated QTLs in *Arabidopsis thaliana*. Plant Cell 27, 969–983. 10.1105/tpc.15.00122 25873386 PMC4558705

[B18] Martinez-Garcia J. F. Monte E. Quail P. H. (1999). A simple, rapid and quantitative method for preparing *Arabidopsis* protein extracts for immunoblot analysis. Plant J. 20, 251–257. 10.1046/j.1365-313x.1999.00579.x 10571885

[B19] Miller G. Stein H. Honig A. Kapulnik Y. Zilberstein A. (2005). Responsive modes of *Medicago sativa* proline dehydrogenase genes during salt stress and recovery dictate free proline accumulation. Planta 222, 70–79. 10.1007/s00425-005-1518-4 15809861

[B20] Molinari H. B. C. Marur C. J. Daros E. de Campos M. K. F. de Carvalho J. Bespalhok J. C. (2007). Evaluation of the stress-inducible production of proline in transgenic sugarcane (*Saccharum* spp.): osmotic adjustment, chlorophyll fluorescence and oxidative stress. Physiol. Plant 130, 218–229. 10.1111/j.1399-3054.2007.00909.x

[B21] Nanjo T. Kobayashi M. Yoshiba Y. Kakubari Y. Yamaguchi-Shinozaki K. Shinozaki K. (1999). Antisense suppression of proline degradation improves tolerance to freezing and salinity in *Arabidopsis thaliana*. FEBS Lett. 461, 205–210. 10.1016/S0014-5793(99)01451-9 10567698

[B22] Ober E. S. Sharp R. E. (1994). Proline accumulation in maize (*Zea mays* L.) primary roots at low water potentials. 1. Requirement for increased levels of abscisic acid. Plant Physiol. 105, 981–987. 12232259 10.1104/pp.105.3.981 PMC160749

[B23] Parre E. Ghars M. A. Leprince A. S. Thiery L. Lefebvre D. Bordenave M. (2007). Calcium signaling via phospholipase C is essential for proline accumulation upon ionic but not nonionic hyperosmotic stresses in *Arabidopsis*. Plant Physiol. 144, 503–512. 10.1104/pp.106.095281 17369432 PMC1913778

[B24] Peng Z. Lu Q. Verma D. P. S. (1996). Reciprocal regulation of *Δ*^1^-pyrroline-5-carboxylate synthetase and proline dehydrogenase genes controls proline levels during and after osmotic stress in plants. Mol. Gen. Genet. 253, 334–341. 9003320 10.1007/pl00008600

[B25] Savoure A. Hua X. J. Bertauche N. VanMontagu M. Verbruggen N. (1997). Abscisic acid-independent and abscisic acid-dependent regulation of proline biosynthesis following cold and osmotic stresses in *Arabidopsis thaliana*. Mol. Gen. Genet. 254, 104–109. 10.1007/s004380050397 9108297

[B26] Savoure A. Jaoua S. Hua X. J. Ardiles W. Vanmontagu M. Verbruggen N. (1995). Isolation, characterization and chromosomal location of a gene encoding the *Δ*^1^-pyrroline-5-carboxylate synthetase in *Arabidopsis thaliana*. FEBS Lett. 372, 13–19. 10.1016/0014-5793(95)00935-3 7556633

[B27] Sawahel W. A. Hassan A. H. (2002). Generation of transgenic wheat plants producing high levels of the osmoprotectant proline. Biotech. Lett. 24, 721–725. 10.1023/A:1015294319114

[B28] Schertl P. Cabassa C. Saadallah K. Bordenave M. Savoure A. Braun H. P. (2014). Biochemical characterization of proline dehydrogenase in *Arabidopsis* mitochondria. FEBS J. 281, 2794–2804. 10.1111/febs.12821 24751239

[B29] Servet C. Ghelis T. Richard L. Zilberstein A. Savoure A. (2012). Proline dehydrogenase: a key enzyme in controlling cellular homeostasis. Front. Biosci. 17:3947. 10.2741/3947 22201764

[B30] Sharma S. Lin W. D. Villamor J. G. Verslues P. E. (2013). Divergent low water potential response in *Arabidopsis thaliana* accessions Landsberg erecta and Shahdara. Plant Cell Environ. 36, 994–1008. 10.1111/pce.12032 23130549

[B31] Sharma S. Verslues P. E. (2010). Mechanisms independent of ABA or proline feedback have a predominant role in transcriptional regulation of proline metabolism during low water potential and stress recovery. Plant Cell Environ. 33, 1838–1851. 10.1111/j.1365-3040.2010.02188.x 20545884

[B32] Sharma S. Villamor J. G. Verslues P. E. (2011). Essential role of tissue-specific proline synthesis and catabolism in growth and redox balance at low water potential. Plant Physiol. 157, 292–304. 10.1104/pp.111.183210 21791601 PMC3165878

[B33] Skirycz A. Vandenbroucke K. Clauw P. Maleux K. De Meyer B. Dhondt S. (2011). Survival and growth of *Arabidopsis* plants given limited water are not equal. Nat. Biotech. 29, 212–214. 10.1038/nbt.1800 21390020

[B34] Strizhov N. Abraham E. Okresz L. Blickling S. Zilberstein A. Schell J. (1997). Differential expression of two P5CS genes controlling proline accumulation during salt-stress requires ABA and is regulated by ABA1, ABI1 and AXR2 in *Arabidopsis*. Plant J. 12, 557–569. 10.1111/j.0960-7412.1997.00557.x 9351242

[B35] Su J. Wu R. (2004). Stress-inducible synthesis of proline in transgenic rice confers faster growth under stress conditions than that with constitutive synthesis. Plant Sci. 166, 941–948. 10.1016/j.plantsci.2003.12.004

[B36] Szabados L. Savouré A. (2010). Proline: a multifunctional amino acid. Trends Plant Sci. 15, 89–97. 10.1016/j.tplants.2009.11.009 20036181

[B37] Szekely G. Abraham E. Cselo A. Rigo G. Zsigmond L. Csiszar J. (2008). Duplicated P5CS genes of *Arabidopsis* play distinct roles in stress regulation and developmental control of proline biosynthesis. Plant J. 53, 11–28. 10.1111/j.1365-313X.2007.03318.x 17971042

[B38] Tateishi Y. Nakagawa T. Esaka M. (2005). Osmotolerance and growth stimulation of transgenic tobacco cells accumulating free proline by silencing proline dehydrogenase expression with double-stranded RNA interference technique. Physiol. Plant 125, 224–234. 10.1111/j.1399-3054.2005.00553.x

[B39] Trontin C. Tisne S. Bach L. Loudet O. (2011). What does *Arabidopsis* natural variation teach us (and does not teach us) about adaptation in plants? Curr. Opin. Plant Biol. 14, 225–231. 10.1016/j.pbi.2011.03.024 21536479

[B40] Vélez-Bermúdez I. C. Schmidt W. (2014). The conundrum of discordant protein and mRNA expression. Are plants special? Front. Plant Sci. 5:619. 10.3389/fpls.2014.00619 25426129 PMC4224061

[B41] Verslues P. E. Lasky J. R. Juenger T. E. Liu T. W. Kumar M. N. (2014). Genome-wide association mapping combined with reverse genetics identifies new effectors of low water potential-induced proline accumulation in *Arabidopsis*. Plant Physiol. 164, 144–159. 10.1104/pp.113.224014 24218491 PMC3875797

[B42] Verslues P. E. Sharma S. (2010). Proline metabolism and its implications for plant-environment interaction. Arabidopsis Book 8, e0140. 10.1199/tab.0140 22303265 PMC3244962

[B43] Verslues P. E. Sharp R. E. (1999). Proline accumulation in maize (*Zea mays* L.) primary roots at low water potentials. II. Metabolic source of increased proline deposition in the elongation zone. Plant Physiol. 119, 1349–1360. 10.1104/pp.119.4.1349 10198094 PMC32020

[B44] Voetberg G. S. Sharp R. E. (1991). Growth of the maize primary root at low water potentials. 3. Role of increased proline deposition in osmotic adjustment. Plant Physiol. 96, 1125–1130. 10.1104/pp.96.4.1125 16668308 PMC1080903

[B45] Yancey P. H. Clark M. E. Hand S. C. Bowlus R. D. Somero G. N. (1982). Living with water stress: evolution of osmolyte systems. Science 217, 1214–1222. 10.1126/science.7112124 7112124

[B46] Yoshiba Y. Kiyosue T. Katagiri T. Ueda H. Mizoguchi T. Yamaguchi-Shinozaki K. (1995). Correlation between the induction of a gene for *Δ*^1^-pyrroline-5-carboxylate synthetase and the accumulation of proline in *Arabidopsis thaliana* under osmotic stress. Plant J. 7, 751–760. 10.1046/j.1365-313X.1995.07050751.x 7773306

[B47] Yoshiba Y. Kiyosue T. Nakashima K. Yamaguchi-Shinozaki K. Shinozaki K. (1997). Regulation of levels of proline as an osmolyte in plants under water stress. Plant Cell Physiol. 38, 1095–1102. 10.1093/oxfordjournals.pcp.a029093 9399433

[B48] Yoshiba Y. Nanjo T. Miura S. Yamaguchi-Shinozaki K. Shinozaki K. (1999). Stress-responsive and developmental regulation of *Δ*^1^-pyrroline-5-carboxylate synthetase 1 (P5CS1) gene expression in *Arabidopsis thaliana*. Biochem. Biophys. Res. Commun. 261, 766–772. 10.1006/bbrc.1999.1112 10441499

[B49] Zhang C. S. Lu Q. Verma D. P. S. (1995). Removal of feedback inhibition of *Δ*^1^-pyrroline-5-carboxylate synthetase, a bifunctional enzyme catalyzing the first two steps of proline biosynthesis in plants. J. Biol. Chem. 270, 20491–20496. 10.1074/jbc.270.35.20491 7657626

[B50] Zhu B. C. Su J. Chan M. C. Verma D. P. S. Fan Y. L. Wu R. (1998). Overexpression of a *Δ*^1^-pyrroline-5-carboxylate synthetase gene and analysis of tolerance to water- and salt-stress in transgenic rice. Plant Sci. 139, 41–48. 10.1016/S0168-9452(98)00175-7

